# Mammogram Screening Uptake and Its Associated Factors among Female Staff in Health Campus, Universiti Sains Malaysia, Kelantan, Malaysia

**DOI:** 10.21315/mjms2023.30.5.12

**Published:** 2023-10-30

**Authors:** Nurul Aizam Mohd Azmi, Juliawati Muhammad, Siti Suhaila Mohd Yusoff, Nik Rosmawati Nik Hussin

**Affiliations:** Department of Family Medicine, School of Medical Sciences, Universiti Sains Malaysia, Kelantan, Malaysia

**Keywords:** breast cancer screening, mammogram, health staff

## Abstract

**Background:**

Breast cancer is among the most common cancers in Malaysia and around the world. Early detection is essential to improve outcomes, increase survival and reduce the death rate. Breast cancer screening via mammography is one of the proven effective methods. Health staff plays an important role to increase awareness and promote mammogram screening in the community. This study aims to determine the prevalence of mammogram screening and its associated factors among the female staff at Health Campus, Universiti Sains Malaysia.

**Methods:**

A cross-sectional study was conducted among the female staff aged 50 years old and above in Health Campus, Universiti Sains Malaysia. The data were collected using a self-administered questionnaire and the Viarad online system. The questionnaires include sociodemographic information, medical factors, knowledge regarding breast cancer and health beliefs about breast cancer. The Viarad online system was used to trace data of mammogram findings for those who underwent mammogram screening.

**Results:**

Among 260 participants, the prevalence of mammogram screening uptake was only 51.9%. By using statistical analysis simple logistic regression and multiple logistic regression, we found that the most significant associated factors were age, clinical breast examination (CBE), level of knowledge and physician recommendation. The mammogram screening showed that most of the breast cancer findings in Breast Imaging Reporting and Data System (BI-RADS) category 2 were at a rate of approximately 35.6%.

**Conclusion:**

This study showed the prevalence of mammogram screening uptake among the female staff was 51.9% although the service is free, readily available and accessible. The older age group, CBE, physician recommendation and knowledge about breast cancer were the main associated factors for mammogram screening uptake in the female staff in this hospital. An education programme aimed at improving the knowledge and role of a physician in promoting mammogram screening among staff should be established.

## Introduction

Breast cancer is the most common cancer among women worldwide, as well as in Malaysia. Breast cancer accounts for 23% of the total new cancer cases globally (1, 2). Meanwhile, in Malaysia, breast cancer contributed to 19% of the new cancers diagnosed in 2012–2016, accounting for 33.9% of Malaysian women (1). The total cases of women who were diagnosed with breast cancer increased by 18%, that is, from 18,206 to 21,634 cases for the period 2007–2011 and 2012–2016, respectively (3). Breast cancer contributes to the most common causes of death worldwide, accounting for approximately 14% of cancer-related deaths (2). According to National Registration Department in Malaysia, the total number of cancer-related deaths was approximately 82,601 cases from 2012 to 2016 (3). Unfortunately, breast cancer tends to be diagnosed at a later stage, and it is the leading cause of death in Malaysian women. According to the National Cancer Registry, Malaysia reported that in 47.9% of cases, breast cancer was diagnosed in the later stage in 2012–2016 than in 2007–2011 (43.2%) (3). Thus, early diagnosis via mammogram screening can improve outcomes and reduce cancer mortality (1). Additionally, a person diagnosed with breast cancer at an early stage has better chance of survival and prognosis than those diagnosed at a late stage (1).

There are a few breast screening methods, but only mammography has proven to be an effective method for breast cancer screening (4). Similarly, in Malaysia, mammography is a gold standard for breast cancer screening (1). According to the National Guideline, asymptomatic low-risk women aged 50 years old–74 years old undergo mammography every 2 years. Although mammography screening is a free screening for the targeted population, the prevalence of mammography remains low in Malaysia (5).

Few factors were found to be associated with mammogram screening uptake in Malaysia. These include sociodemographic information, medical factors, physician recommendations, knowledge regarding breast cancer and health beliefs about breast cancer (6). Furthermore, health and medical personnel play important role in health promotion, especially in breast cancer screening (7, 8).

According to National Health and Morbidity Survey 2019, the prevalence of mammography among women in Malaysia countries was low, that is, 21%, whereas the states on the east coast of Peninsular Malaysia such as Pahang and Kelantan showed the lowest prevalence, constituting 10.6% and 7%, respectively, when compared with the states in the west coast of Peninsular Malaysia such as Kuala Lumpur, constituting 37.9% (9). By contrast, the study in the state situated in the west coast region, which is conducted in Kuala Lumpur Tertiary Hospital, showed a high prevalence of mammogram screening uptake among the staff, which was approximately 83.5% (7). However, there is no similar study done in the state situated on the east coast of Malaysia. Moreover, the research conducted in Kuala Lumpur tertiary hospital was 11 years ago, and no recent study was performed in a hospital in which mammogram screening is available (7).

This study aimed to determine the prevalence of mammography screening uptake and its associated factors among female staff and to determine the associated factors that contribute to mammogram screening uptake among the female staff in the Health Campus, Hospital Universiti Sains Malaysia, in Kelantan, which is situated on the east coast of Peninsular Malaysia. Hence, these findings can be used for a better understanding of associated factors influencing mammogram uptake. Hopefully, the information from this study will identify the most significant factors and help to improve mammogram screening uptake among female staff specifically and communities generally. Additionally, the staff can influence the general population to participate in breast screening activities and result in the improvement of mammogram screening programmes in communities.

## Methods

This cross-sectional study was conducted among 260 female staff aged 50 years old and above at Health Campus, Universiti Sains Malaysia from 21 June 2021 to 21 June 2022. Female staff with a history of breast cancer, currently seeking breast cancer treatment or having benign breast conditions were excluded.

The sample size was calculated using the single proportion formula. The reference proportion of female staff who underwent mammogram screening, which was 80.3%, was based on a previous study done at tertiary hospital in Kuala Lumpur (7). Taking the precision of 0.05 with 95% confidence, the minimum required sample was 245 and after considering the 10% nonresponder rate, the sample is calculated as 267.

The sampling method used to select the respondent in this study was simple random sampling. Initially, a list of names of the female staff aged 50 years old and above with their office telephone number was obtained from the Registry Department, Universiti Sains Malaysia. Then, simple random sampling was employed, using Microsoft Excel, to obtain 267 respondents.

### Research Tools

To achieve the objective of the study, the research tools used in this study were a self-administered questionnaire and Viarad online systems to trace mammogram findings. The self-administered questionnaire consisted of five sections, which include sociodemographic information, medical factors, health beliefs about breast cancer, knowledge regarding breast cancer and mammogram findings. Section A consisted of sociodemographic information, which includes age, ethnicity, marital status, the highest level of education, occupation and monthly household income. Section B comprised health details in terms of medical factors and included 18 questions on obstetrics and gynaecology and medical illness. It also asked about breast self-examination (BSE), clinical breast examination (CBE), pap smear screening, oral contraceptives and hormone replacement therapy (HRT) use, as well as a family history of breast cancer. Sections C and D included health belief and knowledge questionnaires based on a validated questionnaire in a Malaysian study (5). It is based on the Champion Health Belief Model Scale 1998. Cronbach’s alpha coefficient ranged from 0.778 to 0.958.

The questionnaire regarding belief consisted of 20 items divided into three domains. The three domains were: i) perceived susceptibility (three items), ii) the benefit of a mammogram (five items) and iii) barriers to a mammogram (12 items). The response for these items is on a Likert scale, ranging from strongly disagree (one point), disagree (two points), neutral (three points), agree (four points) and strongly agree (five points). The total score is a summation of the points obtained for all items for each domain. The score was recategorised as strong belief, poor belief and neutral belief using a threshold that is calculated by multiplying 3 (points for answering neutral) by the number of items in each domain. The susceptibility domain was categorised into strong belief (total score more than 9), no belief (total score equal to 9) and poor belief (total score less than 9); the benefit of mammogram was categorised into strong belief (total score more than 15), no belief (total score equal to 15) and poor belief (total score less than 15); and the barrier to mammogram domain was categorised into strong belief (total score more than 20), no belief (total score equal to 20) and poor belief (total score less than 20) (5).

The knowledge questionnaires include 35 items that were divided into four categories: i) breast cancer prevalence (four items), ii) symptoms (seven items), iii) risk factors (13 items) and iv) breast cancer screening and programme (11 items). For each item, one point is awarded if the answer is ‘know’ and zero points if ‘don’t know’. The total score of each domain was determined by adding the scores of its item. The total knowledge score was determined by adding the total scores of each domain. Then, the total knowledge score will be converted into percentages and categorised into levels of knowledge, which are poor (50% and less), intermediate (more than 50%–70%), and good knowledge (more than 70%) (5). The last section, Section E, is a questionnaire on mammography screening. It comprises three questions regarding mammogram practice before and recommendation of a mammogram. The questionnaire was offered in the Malay Language.

The mammogram finding was traced through online tracing (Viarad online system). The findings were classified according to Breast Imaging Reporting and Data System (BI-RADS) assessment categories, which are standardised breast imaging reporting (1).

### Data Collection

The questionnaire was administered between September 2021 and December 2021. A female staff member was identified and then contacted through office telephone numbers. After fulfilling the inclusion and exclusion criteria, a brief explanation of the study was given. The staff members who agreed to join the study were given specific dates, times and places they preferred for questionnaire administration. The Respondent Information Sheet and Consent form with a self-administrated questionnaire were given to the respondents. After reviewing the completed questionnaires, the participants who underwent a mammogram were identified. The data on mammogram findings for those who had done mammograms in USM will be traced via online tracing through Viarad online system.

### Data Analysis

The Statistical Package for Social Sciences Inc, Chicago Illinois version 27.0 was used for data entry and analysis. Descriptive analysis was used to describe the proportion of female staff who underwent mammogram screening and mammogram findings of female staff who underwent mammogram findings. Continuous variables were expressed as mean (SD). Categorical data were presented as frequency and percentage. The dependent variable was mammogram screening status. The independent numerical (continuous) variable was age. The independent categorical variables were marital status, education level, types of occupation, monthly family income, medical illness, family history of breast cancer, BSE, CBE, pap smear screening, oral contraceptive use, physician recommendation, health belief domain and knowledge. Simple and multiple logistic regression analysis was performed for associated factors for undergoing mammogram screening among female staff. The univariate analysis was first performed to select the variables for multiple logistic regressions. The confidence level and level of significance were fixed at 95% and 5%, respectively.

## Results

In total, only 260 (97%) participants completed the questionnaires. Among them, 135 (51.9%) underwent mammogram screening at least once in their life, whereas 125 (48.1%) did not undergo mammogram screening.

The participants aged between 50 years old and 60 years old with a mean (SD) of 54.39 (3.03) years old for those who underwent mammograms and 53.79 (3.01) years old for those who did not. Most of the respondents who underwent mammogram screening were Malay, married, completed tertiary education level, worked as clinical staff members and have family income between RM5,000 and RM10,000, as shown in Table 1.

The result from simple logistic regression presented in Table 2 showed that 13 variables were found to have a value of < 0.3. All statistically significant and clinically significant variables were included in the multiple logistic regression analysis. Table 3 shows multivariate analysis using multiple logistic regression. The Hosmer-Lemeshow test was not significant. The classification table was correctly classified. The multiple logistic regression analysis showed that four factors were significantly associated with mammogram screening uptake, namely, older age, history of CBE, physician recommendation and level of knowledge. There were 1.1 times more participants who underwent mammogram screening with increasing age (OR = 1.11; 95% CI: 1.02, 1.22; *P* = 0.023). A participant who had previous CBE had adjusted odds 4.4 times after undergoing mammogram screening in comparison with those who never had CBE (OR = 4.36; 95% CI: 2.36, 8.05; *P* < 0.001). The participants with physician recommendation 2.1 times underwent mammogram screening (OR = 2.10; 95% CI: 1.21, 3.65; *P* < 0.008). In terms of knowledge level, the participants with intermediate and good knowledge and those with poor knowledge showed 3.2 (OR = 3.24; 95% CI: 1.39, 7.54) and 2.7 (OR = 2.69; 95% CI: 1.19, 6.07) times likely to underwent mammogram screening, respectively.

Among the breast finding results of the 135 participants who underwent mammogram findings, only 127 (94%) were available in Viarad online system, whereas another eight (6%) were not available in this system. This is because some of them do not undergo mammograms in USM. Most of the breast findings in BI-RADS category 2 belong to approximately 48 participants (35.6%), whereas others in the BI-RADS category are shown in Table 4.

## Discussion

Mammogram screening is the main screening in the early detection of breast cancer and the reduction of breast cancer mortality. This study among health staff is important because their experience with mammogram screening can give a positive impact on other women to go for the screening (7, 8, 10).

In our study, the prevalence rate of mammogram screening uptake among participants was 51.9%. The result appears to be higher than the prevalence rate of mammogram uptake in Malaysia, which was 25%, with Kelantan state having the lowest prevalence rate of approximately 7% (9). This highlighted the difference between hospital staff and communities because mammogram screening in our facility is readily available, easily accessible and free for our staff compared to the general population. Contrarily, it was much lower than that in a study conducted in Kuala Lumpur Tertiary Hospital, that is, 83.5% (7). In addition, the impact of the coronavirus disease 2019 (COVID-19) pandemic limits breast screening activity. A similar situation also occurred in a study performed in Taiwan, showing that the number of mammogram screening uptake reduced in comparison with that in the year before the COVID-19 pandemic (11). Moreover, perception and attitude toward primary prevention were different between the state on the east coast and that on the west coast of Peninsular Malaysia, as shown by a study on vaccine perception and refusal in Malaysia (12). However, there was no similar study conducted on screening activities such as a mammogram. There were mixed results about the proportion of health staff who had a mammogram screening at least once in other countries. A similar result was discovered in a study performed among health staff in Palestine, which revealed that 50% of them had mammogram screening, whereas the prevalence rate of mammogram screening uptake among health staff in Saudi Arabia was lower, which was only 18.7% (8, 13). By contrast, mammogram screening uptake is higher in developed countries such as the United States, which showed a high percentage (84%) of health staff went for mammogram screening (14). This is because breast screening guidelines in each country are different (8, 13, 14). Furthermore, the difference is attributed to health promotion and awareness campaigns among the staff and the availability and accessibility of mammogram services in health facilities (7, 8, 15).

The majority of respondents who underwent mammogram screening were Malays, were married, completed tertiary education level, worked as a clinical staff member and have family income between RM5,000 and RM10,000. However, the analysis identifies that age was the only significantly associated factor for mammogram screening uptake.

Age, CBE, physician recommendation and level of knowledge were identified as four significant factors that determine mammogram screening uptake in our study. In our study, the mean age of respondents who underwent mammogram screening was 54.4 years old. There were 1.1 times more respondents who underwent mammogram screening with increasing age. This indicates older women are more likely to go for mammogram screening. Similarly, in a study on mammography screening among Malaysian women attending primary care clinics, those aged between 40 years old and 49 years old were four times more than those aged 50 years old–59 years old and seven times more than those aged 60 years old and above (5). Studies in other countries such as on health workers at university hospital in Turkey showed that older health workers participate more in breast cancer screening because they have more experience when compared with younger health workers (16). Our study selected participants aged 50 years old and above based on the latest Malaysia guideline on breast cancer screening, and mammogram screening offered to women aged 50 years old–74 years old (1). However, a study conducted among health staff in a tertiary hospital in Kuala Lumpur, Malaysia, show no significant results between age and mammography uptake. This difference in age selection in their study as staff aged 40 years old and above were recruited as their participant (7).

Our study revealed that respondents who have had previous CBE were more likely to undergo mammogram findings. Similarly, a study carried out among women who attended clinics in Malaysia revealed that women who had previous CBE had a significant positive predictor for mammography screening uptake (5). Another study done in a suburban district in Malaysia showed that participants who had done CBE were more likely to do mammogram screening. CBE was also identified as one of the significant factors that contribute to mammogram screening uptake in a recent study conducted at Urban University Primary Care Clinic in Malaysia. Moreover, CBE is an opportunity for a clinician to give education to women regarding breast cancer, its symptom, and risk factor and also to teach them about BSE, which increase their awareness of breast cancer (17). A systemic review regarding factors associated with breast cancer screening participation among women in mainland China showed that monthly CBE in women increases the rate of mammogram uptake. Additionally, women who received physical examinations such as CBE are more likely to participate in breast cancer screening (18). Other screening activities such as BSE and pap smear showed an uptake association in mammogram screening. A study found that women who practice monthly BSE increased the rate of undergoing mammograms (18). There was an association between pap smear and mammogram uptake in a study among Canadian women, whereas a study among women in British Columbia revealed that women who had pap smear tests were three times more likely to undergo mammogram screening(15, 19). However, in our study, other screening activities such as BSE and pap smear showed no significant findings.

This study discovered physician recommendations had a positive association with mammogram screening uptake and could be attributed to staff following orders from physicians. Additionally, staff who had more contact with a physician were more likely to undergo screening similar to a study among health personnel in a health facility in Saudi Arabia (13). Furthermore, having regular physician follow-ups has a positive association with attending mammography screening (5, 15). A similar study in a tertiary hospital in Malaysia found that routine examination by a physician and their recommendation in the wellness clinic in the hospital play an important role in encouraging women to go for mammogram screening (7). Moreover, a physician provides more information regarding the procedure to create confidence among women to go for the screening (15). Additionally, it was discovered from other studies that women from lower socioeconomic status and older women were encouraged by their doctors to attend mammogram screening (20). Thus, a physician plays an important role in breast cancer screening, since early detection improves survival and reduces cancer rate.

Knowledge regarding breast cancer and screening plays an important role in mammography screening (21). Our study showed that knowledge has a significant factor for staff for mammogram uptake as participants with intermediate and good knowledge were likely to undergo mammogram screening compared with those who had poor knowledge. Similarly, a systematic review conducted in China revealed that women with a high level of knowledge about breast cancer and the screening method of breast cancer are more likely to undergo screening (18). A study among healthcare workers in Palestine revealed a positive relationship between good knowledge regarding breast cancer and its screening and mammography uptake (8). Additionally, compared to women with good knowledge about breast cancer, those with poor knowledge are less likely to undergo mammography screening (22). Contrarily, a study among the general population in a government primary care clinic in Malaysia and a recent study in primary care clinic in an urban university showed no association between good knowledge and mammography uptake (5, 23).

Our study found that the health belief domain was not a significant risk factor for mammogram screening uptake, which is similar to a study conducted among health staff in a tertiary hospital in Kuala Lumpur (7). However, a literature review of factors that influence breast cancer screening among Asian countries found that health beliefs about the benefit of screening have an association with the screening activity (21). Furthermore, a study in primary healthcare centres among female health personnel showed a health belief that the benefit has a positive association with mammogram screening uptake (8).

Mammogram finding is based on the BI-RADS, which is widely used in breast cancer screening programmes (24). From our study, among 135 respondents who underwent mammogram findings, only 94% of respondents’ breast finding results are available in the Viarad online system, whereas another 6% are not available in this system because they do not undergo mammograms in USM. Most of the breast findings were in BI-RADS category 2, belonging to approximately 48 respondents (35.6%), whereas others were in the BI-RADS category. This indicates that mammogram screening can differentiate benign lesions and malignant lesions and further the stratification of the lesion, which enables earlier referrals and further management (24). Fortunately, in our study, most of the finding is benign, which were in classification BI-RADS 1, 2 and 3, which continue with routine screening for BI-RADS 1 and 2 and need short-interval follow-up for BI-RADS 3 (1). However, there is also a small number in BI-RADS category 4 (2.2%) and 5 (1.7%) detected during a mammogram screening. Even if some participants have a higher risk of malignancy suspicion, earlier detection enables the referral of those individuals for further tissue biopsy and further referral to a specialist (1). It is indicated that mammogram screening is very important in detecting breast cancer earlier because some women are asymptomatic. Thus, by doing mammogram screening, breast cancer can be detected earlier, which allows early treatment and subsequently improves survival.

## Conclusion

To conclude, despite the accessibility and availability of mammogram services and considering health staff, the prevalence rate is still suboptimal. The older age group, CBE, physician recommendation and knowledge were significantly associated with mammogram screening uptake in female staff in this hospital. Interaction between physicians and female staff is important to provide knowledge and recommendation for mammogram screening. Knowledge of breast cancer and breast cancer screening among health professionals is critical for them to undergo screening and, hopefully, influence the community.

Although the response rate was good, however, the current study has some limitations. Despite having a list of female staff names, the researcher is having difficulty in contacting suitable female candidates because some staff work from home during the COVID situation, resulting in the target sample not being met. Since the study was conducted during the COVID-19 pandemic, some of the respondents who have an appointment for a mammogram must be postponed, making the prevalence of mammogram screening much lower than expected.

Due to overreporting or underreporting, self-administered questionnaires may result in information bias. Since the researcher would not be able to access trace findings of mammograms if the participants had done mammogram screening outside of HUSM, the findings of mammograms from HUSM could result in bias of the results. There was also a scarcity of literature on mammogram screening among health staff, particularly in Malaysia.

Given the availability and accessibility of mammogram services, the prevalence rate of 51.9% remains unsatisfactory. The reason for this low prevalence rate is the COVID-19 pandemic, which limited the appointment for mammogram screening. Thus, measures to increase the rate of mammogram screening should be performed after the pandemic has been under control. Our findings in this study can help policymakers such as in hospital administration to improve breast cancer screening promotion and activities. We found that physicians play an important role in providing information and giving advice regarding breast screening activities, especially mammograms. Thus, they should receive updated knowledge regarding current guidelines via continuous medical education. Furthermore, a consistent educational programme for staff should be implemented to increase knowledge about breast cancer and mammograms. This study also explains why CBE should be made more available to staff to increase mammogram screening uptake. Almost half of the participants had never undergone mammogram screening. Thus, further study is required to identify barriers in this population, which contributes to a better understanding of this issue, and hopefully, action will be taken to reduce the modifiable barrier.

## Figures and Tables

**Table 1:** Sociodemographic data on female staff who underwent a mammogram and those who did not

	Did not undergo mammogram		Underwent mammogram	
	
	*n* (%)	Mean (SD)	*n* (%)	Mean (SD)
Age		53.8 (3.0)		54.4 (3.0)
Race				
Malay	121 (49.2)		125 (50.8)	
Non-Malay	4 (28.6)		10 (71.4)	
Marital status				
Married	107 (48)		116 (52)	
Single	18 (48.6)		19 (51.4)	
Education level				
Secondary	59 (57.8)		43 (42.2)	
Tertiary	66 (41.8)		92 (58.2)	
Occupation				
Non-clinical	44 (62)		27 (38)	
Clinical	81 (42.9)		108 (57.1)	
Family income (RM)				
< 5,000	51 (59.3)		35 (40.7)	
5,000–10,000	66 (43.4)		86 (56.6)	
> 10,000	8 (36.4)		14 (63.6)	

Note: SD = standard deviation

**Table 2:** Associated factors for female staff who underwent the mammogram screening via simple logistic regression analysis

Variable	Crude OR^a^	95% CI^b^	Wald stat^c^	*P-*value
Age	1.07	0.99, 1.16	2.54	0.111
Marital status				
Single	1			
Married	0.97	0.49, 1.96	0.01	0.940
Education level				
Secondary	1			
Tertiary	1.91	1.16, 3.17	6.35	0.012
Occupation				
Non-clinical	1			
Clinical	2.173	1.24, 3.80	7.40	0.007
Family income (RM)				
< 5,000	1			
5,000–10,000	1.90	1.11, 3.25	5.59	0.19
> 10,000	2.55	0.97, 6.72	3.59	0.58
Present medical illness				
No	1			
Yes	1.85	1.12, 3.047	5.75	0.016
Family history of BC				
No	1			
Yes	3.25	1.25, 8.42	5.87	0.015
Done BSE				
No	1			
Yes	3.69	1.51, 9.02	8.21	0.004
Done CBE				
No	1			
Yes	4.85	2.76, 8.53	30.17	< 0.001
Done pap smear				
No	1			
Yes	2.26	1.27, 4.02	7.62	0.006
Oral contraceptive use				
No	1			
Yes	1.37	0.82, 2.29	1.49	0.223
HRT use				
No	1			
Yes	3.36	0.69, 16.51	2.23	0.135
Physician recommendation				
No	1			
Yes	2.87	1.73, 4.77	16.76	< 0.001
Susceptibility				
Poor belief	1			
No belief	0.66	0.28, 1.55	0.91	0.341
Strong belief	1.29	0.63, 2.65	0.49	0.484
Benefit of MMG				
Poor belief	1			
No belief	4.00	0.39, 41.23	1.36	0.244
Strong belief	7.15	1.57, 32.61	6.44	0.011
Barrier of MMG				
Strong belief	1			
No belief	1.41	0.37, 5.36	0.25	0.618
Poor belief	2.09	1.06, 4.11	4.57	0.033
Level of knowledge				
Poor	1			
Intermediate	3.60	1.65, 7.85	10.38	0.001
Good	3.47	1.63, 7.38	10.47	0.001

Note: BMI = body mass index; RM = Ringgit Malaysia; BC = breast cancer; BSE = breast self-examination; CBE = clinical breast examination; MMG = mammography screening;

a Crude odds ratio;

b Confidence interval;

c Wald statistic

**Table 3:** Associated factors for female staff who underwent the mammogram screening via multiple logistic regression analysis

Variable	Adjusted OR^a^ (95% CI^b^)	Wald stat c	*P-*value
Age	1.11 (1.02, 1.22)	5.13	0.023
Done CBE			
No	1		
Yes	4.36 (2.36, 8.05)	22.15	< 0.001
Physician recommendation			
No	1		
Yes	2.10 (1.21, 3.65)	6.98	0.008
Level of knowledge			
Poor	1		
Intermediate	3.24 (1.39, 7.54)	7.47	0.006
Good	2.69 (1.19, 6.07)	5.68	0.017

Note: CBE = clinical breast examination;

a Crude odds ratio;

b Confidence interval;

c Wald statistic; Constant = 1.080. Forward and backward LR method was applied; No multicollinearity and no interaction; Hosmer-Lemeshow test not significant (*P* = 0.724); Classification table = 70% was correctly classified; Area under the receiver operating characteristics (ROC) curve = 75.8%; The model fitted well; Model assumptions were met

**Table 4:** Description of breast finding according to the BI-RADS category

BI-RADS category	*n* (%)
BI-RADS 0	20 (14.8)
BI-RADS 1	37 (27.4)
BI-RADS 2	48 (35.6)
BI-RADS 3	18 (13.3)
BI-RADS 4	3 (2.2)
BI-RADS 5	1 (7)
